# Encapsulation of Dyes in Luminescent Metal-Organic Frameworks for White Light Emitting Diodes

**DOI:** 10.3390/nano11102761

**Published:** 2021-10-18

**Authors:** Zhihong Sun, Aaqib Khurshid, Muhammad Sohail, Weidong Qiu, Derong Cao, Shi-Jian Su

**Affiliations:** 1 State Key Laboratory of Luminescent Materials and Devices, School of Chemistry and Chemical Engineering, South China University of Technology, Guangzhou 510640, China; sunzhihong7@163.com (Z.S.); wdddqiu@foxmail.com (W.Q.); 2 Department of Chemistry, University of Sargodha Sub Campus Bhakkar, Bhakkar 30000, Pakistan; chemistuos22@gmail.com (A.K.); msohail91147@gmail.com (M.S.)

**Keywords:** metal–organic frameworks, organic dye, luminescence, white light emitting diodes

## Abstract

The development of white light emitting diodes (WLEDs) holds great promise for replacing traditional lighting devices due to high efficiency, low energy consumption and long lifetime. Metal-organic frameworks (MOFs) with a wide range of luminescent behaviors are ideal candidates to produce white light emission in the phosphor-converted WLEDs. Encapsulation of emissive organic dyes is a simple way to obtain luminescent MOFs. In this review, we summarize the recent progress on the design and constructions of dye encapsulated luminescent MOFs phosphors. Different strategies are highlighted where white light emitting phosphors were obtained by combining fluorescent dyes with metal ions and linkers.

## 1. Introduction

White light emitting diodes (WLEDs), as solid-state lighting sources, have attracted increasing attention in the past decades owing to their potential applications in displays and lighting [1]. WLEDs are energy saving and environmentally friendly, and have higher luminous efficiency than conventional incandescent and fluorescent lamps [2]. Moreover, WLEDs emit polychromatic light rather than monochromatic light that was emitted by traditional light emitting diodes (LEDs) [3]. It is well known that white light can be generated by mixing primary colors (red, green and blue) in appropriate proportions or using a pair of complementary colors [4]. Light sources with Commission International de l′Eclairage (CIE) coordinates (0.33, 0.33), color correlated temperature (CCT) between 2500 K and 6500 K, and color rendering indices (CRI) value above 80 are preferred for high-quality white light illumination [5]. Quantum yield (QY) is another important photophysical parameter, which refers to the ratio of photons emitted to the photons absorbed (unless otherwise specified, QY in this review is the absolute quantum yield). Currently, there are mainly two approaches to produce WLEDs: (1) multichip combination, in which three LEDs with primary colors are mixed appropriately to generate white light [6] and (2) phosphor-converted WLEDs (pc-WLEDs) approach, where phosphors are excited by a single-chip LED to produce white light. For pc-WLEDs, white light can often be obtained by a blue LED coated with a yellow-emitting phosphor or a ultraviolet (UV) LED coated with mixing phosphors [7]. Most commercially available WLEDs are pc-WLEDs due to the high cost and poor color stability of the color-mixed LEDs [8]. The first commercial WLED was developed by Nichia Chemical Co. in 1996 [9], which adopted a blue LED (InGaN) with yellow-emitting phosphor (YAG:Ce). Since then, tremendous progress has been made and the luminous efficacy has increased from 5 lm/W to over 300 lm/W [3]. Phosphors are of vital importance in determining the optical properties of WLEDs, including luminous efficiency, chromaticity coordinates, color temperature, lifetime and reliability. WLEDs phosphors should have the following properties: strong light absorption, broad excitation spectrum, useful emission spectrum, high quantum efficiency, optimal Stokes shift, high stability, etc. [4]. Current phosphors are almost all based on rare-earth metals and their self-quenching and absorption effects lower the phosphor performances [10]. Therefore, it is urgent to develop new phosphors, especially organic luminescent phosphors.

Metal-organic frameworks (MOFs) are a class of porous crystalline materials composed of inorganic and organic moieties via coordination bonds, which are known for tunable pore size, high surface areas, structure flexibility and multiple functionality. These extraordinary properties have made MOFs ideal candidates for catalysis, gas storage and separation, membranes, biomedical imaging and luminescence-based sensing and lighting [11,12]. Specially, MOFs offer a unique platform for the development of luminescent materials due to structural predictability, multifunctionality, nanoscale processability and well-defined environments for luminophores in crystalline states [13,14]. Luminescence in MOFs can arise from organic ligands, metal ions and charge transfers such as ligand-to-metal charge transfer (LMCT), metal-to-ligand charge transfer (MLCT), ligand-to-ligand charge transfer (LLCT) and metal-to-metal charge transfer (MMCT) [15]. In addition, some guests introduced into MOFs via supramolecular interactions can emit or induce luminescence, and white light can be easily obtained by rational structure design and luminescent guest selection. Overall, these various effects have naturally led to speculation that MOFs could find potential applications in WLEDs. The first attempt to obtain white light by using MOFs can be traced back to 2007 [16]. Since then, different color-emitting lanthanide metals, conjugated organic ligands and guest species such as dye molecules and quantum dots have been incorporated in MOFs to generate white light [17,18].

Encapsulation of emissive organic dyes is quite a simple way to obtain MOFs with multiple luminescence emissions [19]. Organic dyes are probably the most widespread fluorophores among the luminescent materials because of wide excitation band, large absorption coefficient, moderate-to-high quantum yields, short fluorescent lifetime and great availability [20]. However, there are two serious problems when directly applying organic dyes in WLEDs. One is the aggregation caused quenching (ACQ) effect induced by π-π stacking interactions of the organic dyes, which results in low fluorescence intensity in solid states in comparison with their bright solution states. Additionally, the other is the thermal and photo-stability of organic dyes [10]. MOFs are ideal supporting materials to prevent organic dyes aggregating in solid states [21,22], since MOFs are highly porous and able to encapsulate molecular dyes in confined pores, so they are capable of preventing aggregation-induced quenching and restricting internal molecular motions to inhibit non-radiative relaxation [23]. In addition, by carefully choosing fluorescent linkers and organic dyes, MOFs can serve as an antenna to transfer energy to the dyes. The emissions from encapsulated dyes can be easily adjusted by changing the component and content of dyes. Moreover, diverse luminescence properties can be achieved by engineering interactions between dyes and constituents of MOFs. Thus, encapsulation of dyes into MOFs is massively proposed as phosphor converters in white light emitting diodes [21].

There are three major methods to encapsulate organic dyes in MOFs [21]. The first is the two-step synthesis method, in which the pristine MOF is synthesized first and then immersed in a solution of fluorescent dyes. Despite the simplicity of this approach, the mismatch size between MOF aperture and organic dyes not only restricts the choice of dyes, but also causes guest leakage, which hiders the extensive application of this approach. The second is the in situ encapsulation method, where dyes are introduced during the crystal formation. Although this method is helpful in obtaining fluorescent MOFs with uniform distribution of fluorescent dyes, more factors including pore size, pore windows and structures of MOFs for desired organic dyes should be considered. The final method is to use fluorescent linkers incorporated in the frame of MOFs, in which permanent fluorescence can be easily obtained, although the steric hindrance caused by bulky ligands often reduces the yield of the fluorescent of MOFs. In practice, fluorescent ligands are often combined with dyes to induce dual emissions, and the ligand-to-dye energy transfer process can be controlled by changing the excitation energy.

MOFs materials with porosity, multifunctionality and crystallinity have aroused much interest since the debut of the “metal-organic frameworks” concept in 1995 [24], and the scope of this research has expanded from structure design and topology analysis to a wide range of applications in gas storage, catalysis and biomedicine [11,12,25,26,27,28]. A number of excellent reviews have summarized the properties and applications of luminescent metal–organic frameworks (LMOFs) [8,10,13,14,15,17,19,20,29,30,31,32], while the reviews that specifically and systemically discuss the encapsulation of organic dyes in MOFs (dye@MOFs) for WLEDs applications are still rare. This review mainly summarizes recent progress achieved in developing pc-WLEDs based on dye@MOFs, where white light can be generated by coating the dye encapsulated MOF hybrids on the corresponding blue-LED chip or UV-LED chip. The emphasis was put on the white light emitting phosphors fabrication. The origin of luminescence in dye@MOFs has been discussed to tune high-quality white light.

## 2. Phosphors Excited by Blue-LED Chip

The combination of a blue-LED chip with yellow phosphors belongs in a partial conversion. The blue light emitting from LED chip is partially absorbed by the phosphor and refurbished into yellow light, while the remaining part of blue light is transmitted through the phosphor [3]. The blue and yellow light, as a pair of complementary colors, mix together to generate white light. Generally, compared to the UV chip WLEDs, the blue LED chips have higher theoretical efficiency, better reproducibility and lower input energy, so they are quite attractive for low-cost bright white-light sources [33]. However, these WLEDs often show low CRI and high CCT caused by red emitting deficiency, which limits their indoor use. In the past decades, the design and synthesis of new blue-light-excitable single-phase phosphors have emerged as a hot research area, and much progress has been made in improving color-rendering properties, especially benefiting from the development of MOF materials. From a fundamental point of view, the abundant luminescent behaviors and ordered structures of MOFs allow for the fine-tuning of emission color across the CIE diagram and improve luminescent intensity simultaneously.

An effective way to improve color-rendering properties is to broaden the emission spectra. Qian et al. [34] simultaneously encapsulated green-emitting coumarin 6 (Cou-6), yellow-emitting rhodamine 6G (R6G) and red-emitting rhodamine 101 (R101) into a MOF crystal to synthesize a yellow broadband phosphor ZJU-28⊃Cou-6/R6G/R101 via ion exchange method. By coating the single-phase phosphor ZJU-28⊃Cou-6/R6G/R101 on commercial blue LED chips, the WLED lamp exhibits bright white light with luminous efficiency of 126 lm/W, CRI of 88 and CCT of 4446 K, and the total quantum yield (QY) can reach up to 82.9%. The good performance was ascribed to the high intrinsic quantum yields of dyes and fluorescence resonance energy transfer (FRET) process between them. In addition, the confinement effects of the MOFs can effectively inhibit the ACQ of dye molecules.

WLEDs can also be fabricated by combining blue chips with dye@MOFs and other commercialized phosphors [35]. Various concentration of rhodamine (Rh) dye was adopted to synthesize a series of Rh@bio-MOF-1 via cation exchange, and then the mixtures of the yellow-emitting Rh@bio-MOF-1, green (Ba,Sr)_2_SiO_4_:Eu^2+^ and red CaAlSiN_3_:Eu^2+^ were coated on the blue LED chip to form phosphor film, which exhibits high luminous efficacy of 94–156 lm/W, CRI of 80–94 and excellent stability.

Unlike ion exchange, in situ encapsulation, in which fluorescent dyes are incorporated into the pores during the preparation of MOF crystals, have the advantage of uniform distribution of the fluorescent molecules, as long as the dyes can stand the synthesis conditions of MOFs. Li et al. [36] adopted the in situ encapsulation approach to avoid tedious ion-exchange synthesis and prevent dye leakage. Two yellow-emitting nanocomposites R6G@ZIF-8 and DBNT@UiO-66 with solubility compatibility and solution processability were synthesized, which can be excited by blue light to generate white light with absolute quantum yield of 63.1% and 22.7%, respectively. Similarly, Qian et al. [37] incorporated red, green and blue dyes into ZIF-8 by in situ self-assembly process to fabricate stable remote-type incandescent white-light device. They evaluated the thermostability and photostability of TPU-encapsulated ZIF-8⊃pm546/pm605/SRh101 phosphor in detail, and found the stability was greatly enhanced with TPU coating, which was ascribed to the protection against the oxygen and water invasion.

Guest species like carbon dots with strong resistance to irradiation and heat are preferable in order to improve the stability of phosphors. Li et al. [38] encapsulated both green-emitting carbon quantum dots (CQDs) and red-emitting rhodamine B (RhB) into ZIF-8, where RhB molecules can be sensitized by CQDs. Single-phase single-shell CQDs&RhB@ZIF-8^2^ and single-phase multi-shell CQDs@ZIF-8^2^@RhB@ZIF-8^2^ were fabricated as yellow phosphors. Multi-shell CQDs@ZIF-8^2^@RhB@ZIF-8^2^ shows higher luminescence efficiency due to a large spatial distance that can suppress the FRET interactions between CQDs and dyes. Benefiting from the host-guest shielding effect, the stability of hybrid materials can be further improved. Tan et al. [39] conducted a long-term material stability test on dye-encapsulated MOF Gaq3@ZIF-8, and the results showed that after 8 months, not only the structure of ZIF-8 could remain stable with Gaq3 dye being encapsulated, but also the absolute QY of Gaq3@ZIF-8 (15%) was exactly the same as prepared, which demonstrates that when trapping Gaq3 in ZIF-8 pores, the host can act as a shield to protect Gaq3 from photodegradation. A WLED emitting uniform white light could be obtained by coating Gaq3@ZIF-8 on a blue LED.

## 3. Phosphors Excited by UV-LED Chip

For WLEDs based on UV-LED and phosphors, all radiation from UV LED is converted into red, green and blue (RGB) light, which refers to full conversion. The phosphors excited by UV-LED chip must emit white light, so RGB phosphors are often adopted. As mentioned before, pc-WLEDs fabricated by blue LED coated with yellow phosphors may suffer such weaknesses as poor CRI and low stability of color temperature, due to deterioration of the chip or the phosphors. By contrast, UV-LED combined with mixed phosphors is one of the best approaches to generate white light for both high luminous efficiency and high CRI, at the expense of poorer efficacy owing to higher wavelength-conversion losses. Recently, developing single-phase white light phosphors is of great significance and different strategies have been adopted to improve UV-LED luminous efficacy. In general, luminescence in MOFs can be obtained from linkers, framework metal ions, and absorbed guests [29].

### 3.1. Luminescence from Organic Dyes

**Figure 1:**
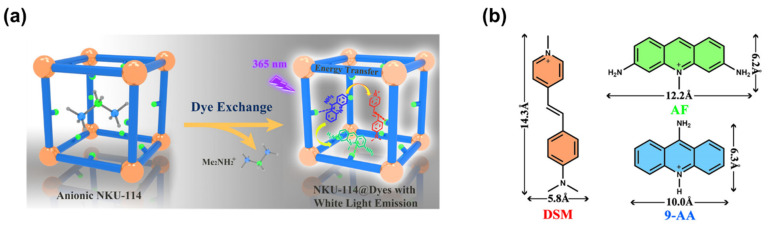
(**a**) Schematic representation of the incorporation of multicomponent dyes in an anionic MOF; (**b**) structures of cationic dyes. (Reproduced with permission from ref. [40]. Copyright © 2020, American Chemical Society).

In order to obtain a single-phase white light phosphor, Bu et al. [40] reported the encapsulation of RGB dyes into anionic MOF via dye exchange, as shown in Figure 1a. NKU-114 with abundant nitrogen sites can serve as an excellent host matrix to incorporate electron-deficient cationic dyes due to the donor–acceptor electrostatic interactions. DSM, AF and 9-AA, with strong red, green and blue light emissions, respectively, were used as selected cationic dyes (Figure 1b), because of suitable spectral overlap and proper molecule size. By carefully tuning the relative contents of three dyes, white-light-emitting NKU-114@DSM-AF/9-AA composite could be obtained, with CIE coordinates (0.34, 0.32), CRI and CCT values of 81 and 5101 K, respectively. The absolute quantum yield reaches a comparative high value of 42.07%, compared with some other reported dye-encapsulated system [41,42]. A WLED was assembled by coating NKU-114@DSM-AF/9-AA on the surface of a UV-LED chip. Under 50 mA, the WLED shows corresponding CIE coordinates, CRI, CCT and luminous efficiency values of (0.3402, 0.3365), 85.41, 5148 K and 2.4 lm/W, respectively.

**Figure 2:**
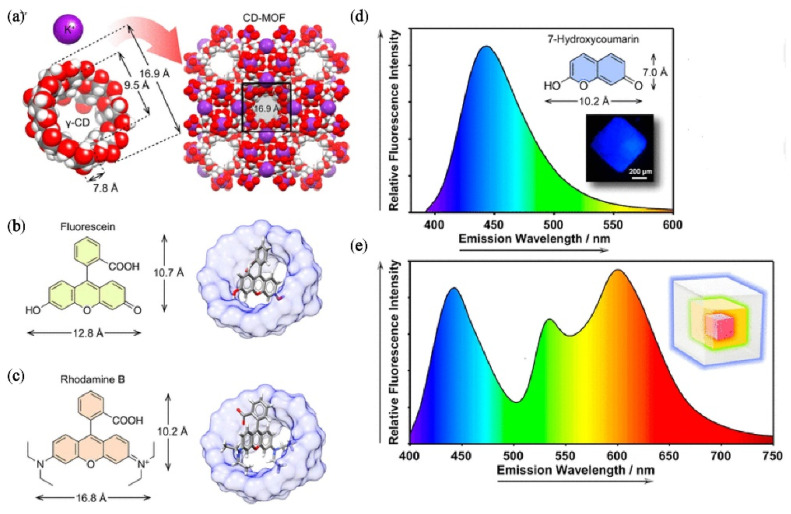
(**a**) γ-CD and formation of CD-MOF; (**b**) structure of FL; (**c**) structure of RhB; (**d**) fluorescence emission spectrum of CD-MOF⊃7-HCm; (**e**) fluorescence emission spectrum of CD-MOF⊃7-HCm@FL@RhB under an excitation wavelength of 365 nm. (Reproduced with permission from ref. [41]. Copyright © 2019, American Chemical Society).

In 2019, inspired by the extensive applications of core-shell structured MOFs, Gong [41] proposed a novel approach to construct core-shell structured cyclodextrin (CD) based MOFs by encapsulating different guests hierarchically into the framework. CD has the ability to improve fluorescence of organic dyes, because it can provide a confined hydrophobic cavity to change the stacking patterns of organic dyes and decrease the freedom of molecular motions [43]. CD-MOF⊃dyes, with γ-cyclodextrin (γ-CD) as organic ligands (Figure 2a), exhibit extremely high luminous intensity because of synergistic effect of CD and MOFs. Fluorescein (FL), RhB and 7-hydroxycoumarin (7-HCm) were chosen as encapsulates. The chemical structures of FL, RhB and fluorescence emission spectrum of CD-MOF⊃7-HCm are shown in Figure 2b–d. CD-MOF⊃RhB with longer emission wavelength was selected as core, and CD-MOF⊃7-HCm@FL@RhB was fabricated via epitaxial seeded growth (Figure 2e). The prepared core-shell crystals emit bright white light upon excitation of UV-LED, with CIE coordinate of (0.35, 0.32).

**Figure 3:**
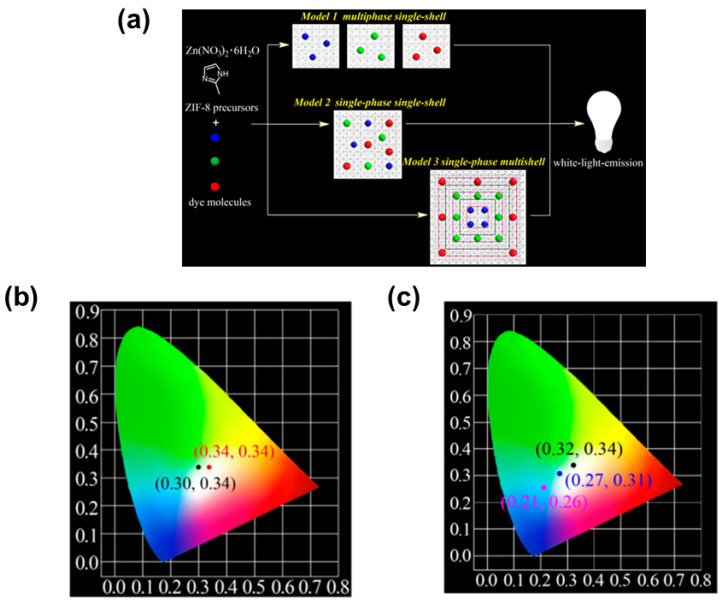
(**a**) Model 1 (multiphase single-shell dye@ZIF-8), Model 2 (single-phase single-shell dyes@ZIF-8), and Model 3 (single-phase multi-shell dyes@ZIF-8); (**b**) CIE coordinates of C-151&F&RB@ZIF-8^2^ with different concentrations of dyes at λ_ex_ = 365 nm; (**c**) CIE chromaticity coordinates of C-151@ZIF-8^2^@F@ZIF-8^2^@RB@ZIF-8^2^ with different concentrations of RB. (Reproduced with permission from ref. [44]. Copyright © 2019, American Chemical Society).

Li et al. [44] reported high quality white-light-emitting dyes@ZIF-8 composites based on the three models (multiphase single-shell dye@ZIF-8, single-phase single-shell dyes@ZIF-8, and single-phase multi-shell dyes@ZIF-8) (Figure 3a), in which dye locations are tunable. Red-emitting rhodamine B (RB), green-emitting fluorescein (F) and C-151 were chosen to match the pore structure of ZIF-8. Multiphase single-shell dye@ZIF-8^2^ is solution-processable for device fabrication, and white light can be generated by optimizing the ratio of C-151@ZIF-8^2^, F@ZIF8^2^, and RB@ZIF-8^2^, with CIE coordinates of (0.32, 0.34). Single-phase single-shell C-151&F&RB@ZIF-8^2^ composite can be prepared by introducing C-151, F, and RB simultaneously into ZIF-8 via in situ encapsulation. By carefully tuning the content of red, green and blue emitting dyes, white light emitting C-151&F&RB@ZIF-8^2^ composites with CIE coordinates (0.30, 0.34) and (0.34, 0.34) were obtained (Figure 3b). The efficiency decrease problem caused by FRET process can be solved by adopting model 3, a single-phase multi-shell dyes@ZIF-8. In model 3, RB, F and C-151 were encapsulated successively into ZIF-8 using shell-by-shell overgrowth, and the CIE chromaticity coordinates of multi-shell C-151@ZIF-8^2^ @F@ZIF-8^2^ @RB@ZIF-8^2^ changed from (0.21, 0.26) to (0.32, 0.34) by tuning the concentration of RB (Figure 3c).

### 3.2. Luminescence from Dyes and Metals

During the development of luminescent MOFs, the lanthanide MOFs have aroused extensive interest from the very beginning owing to high luminescence quantum yield, large Stokes shifts and sharp line-emissions [30]. Since f–f transition is parity-forbidden, lanthanide ions are often sensitized by organic ligands due to antenna effect. Qian [45] fabricated a phosphor for WLED by encapsulating blue dye within lanthanide MOF. EuBPT, TbBPT and Eu_x_Tb_y_BPT were synthesized by the solvothermal reaction. Owing to the energy transfer from BPT ligands to the lanthanide ions, the absolute quantum yields of red-emitting EuBPT and green-emitting TbBPT reached 37.11% and 73.68%, respectively. By optimizing the Eu^3+^/Tb^3+^ ratio, Eu_0.05_Tb_0.95_BPT exhibits yellow light, and when combined with blue dye C460, white light emitting phosphor with absolute QY of 43.42% could be generated. The CRI and CCT values of the phosphors were estimated to be 90 and 6034 K, respectively. The WLED devices were fabricated by coating the prepared phosphor on a commercial UV-LED chip, and the luminous efficiency was measured to be 7.9 lm/W. Similarly, Saha [46] incorporated a single red emitting dye RhB into blue emitting gadolinium-based MOF to achieve perfect white light with high quantum yield.

Apart from the lanthanide, actinide can also be used to construct luminescent MOFs. Recently, inspired by the concept of ‘molecular compartment’ [47], Luo et al. synthesized a cage-based actinide MOF ECUT-300 [48]. Due to the trigonal building unit being constructed from the coordination of uranyl ions and carboxylate, ECUT-300 with mesopore A (2.8 nm), mesopore B (2.0 nm) and micropore C (0.9 nm) could be fabricated. Combining uranyl ions and 4,4′,4′′,4′′′-(ethene-1,1,2,2-tetrayl)tetrabenzoic acid as ligand, ECUT-300 with blue-green emission was observed upon excitation at 408 nm. Interestingly, RhB was encapsulated in the cage B of ECUT-300, and WLED device could be fabricated by coating RhB@ECUT-300 on an UV LED. While [Fe(tpy)^2^]^3+^ was encapsulated in cage C, which could be used to selectively adsorb C_2_H_2_ over CO_2_. In addition, the incorporation of both RhB^+^ and [Fe(tpy)^2^]^3+^ is helpful in stabilizing the framework structure.

### 3.3. Luminescence from Dyes and Organic Linkers

Combining the emissions from linkers and dyes to generate single-phase white light phosphors is a hot research topic in recently years. In 2015, Qian [49] first encapsulated two dyes simultaneously into blue-emitting anionic MOFs via ion exchange. ZJU-28 exhibits blue emission under excitation at 365 nm, which ascribes to the H_3_BTB ligand. ZJU-28⊃DSM/AF, as white lighting phosphor, can be easily prepared by soaking ZJU-28 into the mixed solution of red-emitting DSM and green-emitting AF, exhibiting broadband white emission with CIE coordinates of (0.34, 0.32), CRI value up to 91% and CCT of 5327 K. Since the confinement of MOFs can effectively suppress ACQ, the absolute QY could be improved to 17.4%. Substituting the H_3_BTB ligand with carbazole-based ligand 4,4′,4′’-(9*H*-carbazole-3,6,9-triyl)-tribenzoic acid (H_3_L) [50], a white-light-emitting phosphor with same CRI value could be obtained, while the quantum yield could reach up to 39.4%. 

**Figure 4:**
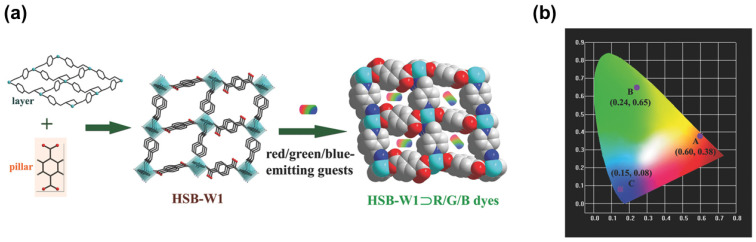
(**a**) Schematic synthesis of HSB-W1⊃R/G/B Dyes. (**b**) The CIE coordinates of HSB-W1⊃DCM (DCM, 0.31 wt%), HSB-W1⊃C6 (C6, 0.04 wt%), and HSB-W1⊃CBS-127 (CBS-127, 0.03 wt%) (λ_ex_ = 365 nm). (Reproduced with permission from ref. [51]. Copyright © 2019 Wiley—VCH Verlag GmbH & Co. KGaA, Weinheim).

It is worth noting that efficient blue emission plays an important role in developing WLED, so strong blue fluorescent molecules are often introduced in company with red and green fluorescent molecules. Zhu [51] reported the incorporation of neutral and ionic RGB guest molecules into a neutral MOF HSB-W1 (Figure 4a). HSB-W1 exhibits blue emission upon excitation at 365 nm. HSB-W1⊃DCM, HSB-W1⊃C6 and HSB-W1⊃CBS-127 can be conveniently synthesized and exhibit red, green and blue emission, respectively, as shown in (Figure 4b). HSB-W1⊃DCM⊃C6⊃CBS-127 composite emits white-light with high quantum yield (up to 26%) and CRI (up to 92%). The results showed that incorporating RGB dyes into blue-emission MOFs is a useful strategy to design single-phase white-light phosphors.

The combination of blue and yellow emission can generate white light. Apart from H_3_BTB and HSB ligands, 9,10-dibenzoate anthracene (DBA) is also an efficient blue emitter. A new phosphor for WLED was fabricated by encapsulating RhB into Al-DBA, and exhibits an emission lifetime of 1.8 ns and 5.4 ns for the blue and yellow light, respectively, enabling the WLED for visible light communication (VLC) [23].

There is a class of materials that are non-emissive in dilute solutions, but become highly luminescent after aggregation. This phenomenon is termed “aggregate induced emission” (AIE), which was proposed by Tang [52]. Tetraphenylethylene (TPE) as a typical AIE luminogen is of great interest in WLEDs [53]. Fu et al. [54] synthesized a TPE-based MOF with broadband green-yellow emission due to the energy transfer between dual linkers. White light can be generated by encapsulation of sulforhodamine 101 (SR101) into the MOF matrix, with corresponding QY, CRI and CCT values of 41.7%, 81.3 and 4527 K, respectively. Zhou et al. [55] investigated the dye encapsulation in TPE-based MOF for WLED. PCN-128W containing TPE-based ligand can be used to sensitize dye molecules through FRET, so DSM@PCN-128W shows dual emissions upon a single excitation. Compared with PCN-128W, the H_4_ETTC ligand exhibits about 70 nm red-shift, because the confinement of MOFs increases the HOMO-LUMO energy gap of the linkers and generates high energy emission. In order to understand better, the typical molecular structures of the ligands are summarized in Table 1. By coating DSM@PCN-128W on UV LED chip, a WLED was obtained showing CIE chromaticity coordinates of (0.34, 0.33), CRI of 79.1, and CCT of 5525 K, and the absolute quantum yield of DSM@PCN128W was measured to be 21.2%. Similarly, by substituting H_4_ETTC with H_8_ETTB as a carboxylate ligand, Dong and Lei synthesized PCN-921 with a strong fluorescence emission at 447 nm [56]. The innovation of their work lies in the realization of room-temperature phosphorescence and white light emission by hierarchically encapsulating coronene and RhB dye. The results showed that by introducing guests into MOFs, coronene@PCN-91 exhibited a phosphorescence lifetime of 62.5 ns, and the hybrid material RhB@coronen@PCN-921 emitted bright white light by coating on a commercial UV LED. In addition, some TPE-based luminescent MOFs also exhibit piezofluorochromic behavior, and by combining organic dye encapsulation, white-light emission can be obtained. Adopting this strategy, Pan and coworkers constructed dual-emission luminescent MOF HNU-49 and generated relative pure white light by adjusting the pressure and the concentration of RhB [57]. The key parameters for white LEDs with dye-encapsulated MOFs as phosphors are summarized in Table 2 for easy comparison.

**Figure 5:**
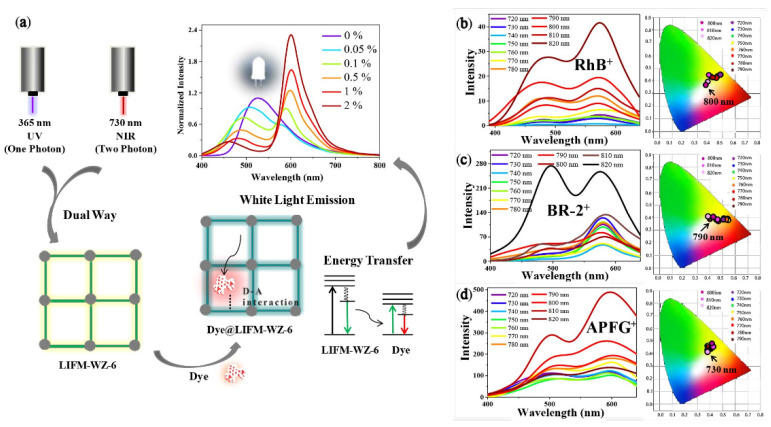
(**a**) Schematic illustration of OPA and TPA dual-way excited white-light emission in dye@LIFM-WZ-6; (**b**) TPEF spectra and CIE coordinate values of RhB+@LIFM-WZ-6 (0.05 wt%); (**c**) TPEF spectra and CIE coordinate values of BR-2+@LIFM-WZ-6 (1 wt%); (**d**) TPEF spectra and CIE coordinate values of APFG+ @LIFM-WZ-6 (0.05 wt%). (Reproduced with permission from ref. [42]. Copyright © 2019 Wiley—VCH Verlag GmbH & Co. KGaA, Weinheim).

The luminescent MOFs mentioned above exhibit a large Stokes shift to prevent self-absorption, which are referred to as down-conversion materials. Another type of MOFs belongs to up-conversion materials, exhibiting an anti-Stokes shift luminescence character. Generally, two methods can be used to achieve up-conversion in MOFs, one exploiting energy transfer between lanthanide ions, the other triplet-triplet annihilation, which is based on ligand selection and design [20]. In 2019, Pan reported a series of dye-encapsulated MOFs exhibiting dual way (one-photon absorption (OPA) and two-photon absorption (TPA)) excited fluorescence (Figure 5a) [42]. LIFM-WZ-6 containing TPE ligand shows blue-green and strong green emission upon excitation at 365 nm and 730 nm, respectively. Electron-deficient cationic dyes RhB^+^, BR-2^+^, BR-46^+^, DSM^+^ and APFG^+^ were chosen due to appropriate D-A interactions and molecule size. RhB^+^@LIFM-WZ-6 was first synthesized and when excited at 365 nm, the corresponding CIE coordinates, CCT, CRI and absolute QY values were (0.33, 0.35), 4745 K, 77 and 9.8%, respectively. Similar results were obtained for another four dyes, confirming the universality of the OPA approach. WLED devices were fabricated by coating the prepared phosphors on the surface of the commercial UV LED chip. Compared with the typical OPA process, two-photon excited fluorescence emission (TPEF) is more complicated and often shows different colors. Through TPA process, white light emitting phosphors RhB^+^@LIFM-WZ-6, BR-2^+^WZ-6 and APFG^+^@LIFM@LIFM-WZ-6 were obtained under the excitation of 800, 790 and 730 nm, respectively (Figure 5b–d). More importantly, the emissive color of dye@MOF can be adjusted by simply tuning the excitation wavelengths.

### 3.4. Organic Dyes as Fluorescent Linkers

**Figure 6:**
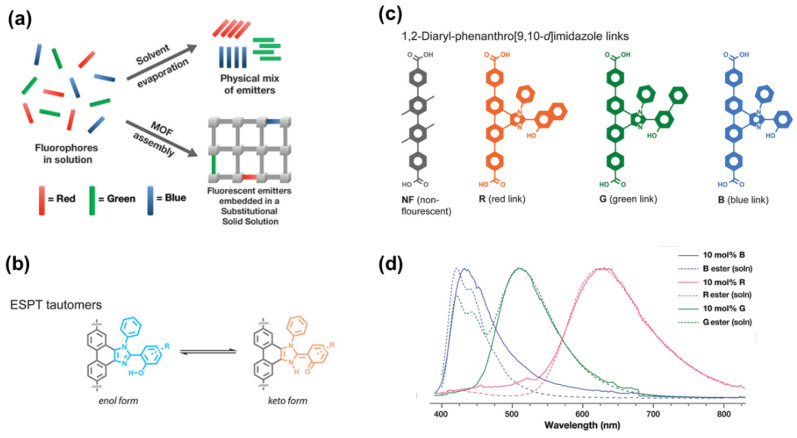
(**a**) Representation of luminescent MOFs based SSS. (**b**) Excited-state proton transfer enol and keto tautomer behavior of dyes. (**c**) Structure of organic linkers. (**d**) Solid-state emission of MOF with 10%-R, 10%-G and 10%-B peaks centered at 430, 510, and 630 nm. (Reproduced with permission from ref. [58]. Copyright © 2019, American Chemical Society).

Inspired by substitutional solid solutions (SSS) concept applied in inorganic materials, Newsome [58] constructed luminescent MOFs by combining nonfluorescent linkers with dilute RGB fluorescent organic dyes, as shown in Figure 6a. Excited-state proton transfer (ESPT) dyes are of extensive interest due to unique photophysical properties caused by keto-enol tautomerism. They have enol tautomers in the ground state, but exists as a keto tautomers after excitation (Figure 6b). Multivariate MOFs are attractive for making multicolor emitting crystals, and the non-fluorescent link and ESPT dyes (Figure 6c) are chosen because of good stability, high quantum yield and color variability. Solid-state emission peaks centered at 430, 510, and 630 nm (Figure 6d) were seen after excitation at 365 nm for 10%-R, 10%-G and 10%-B, respectively. The keto emission in the MOFs is quite close to the ester forms of the RGB links solvated in toluene, as shown in dashed lines, suggesting that prepared MOFs exhibit solution-like properties. Finally, a series of Zr_6_O_4_(OH)_4_(R*_x_*G_1-2*x*_B*_x_*)*_y_*NF_1-*y*_ MOFs were synthesized. (Zr_6_O_4_(OH)_4_(R_0.4_G_0.2_B_0.4_)_0.01_NF_0.99_) emitted combination of broadband emissions from RGB, with coordinates of (0.31, 0.33) on the CIE chromaticity diagram, an absolute QY of 4.3%, a CRI of 93 and a CCT of 6480 K. Other prepared MOFs also exhibited good fluorescence performance. These findings showed that substituting MOF linkers with fluorescent dyes are capable of obtaining both tunable emission chromaticity and accurate color rendering.

Recently, Liu and Li applied a mixed-linker strategy to successfully synthesize a series of UiO-68 MOFs with full color emission by changing the ratios of chromophore and non-fluorescent linkers [59]. Obviously, introducing of non-fluorescent linkers is helpful in reducing the concentration of emissive linkers and increasing the spatial distances between fluorescent linkers, which effectively suppresses the π-π stacking interactions and thus enhances the emission efficiency. It is believed that this general approach is of great significance to overcome the challenge of ACQ, portending the potential application of luminescent MOFs in WLEDs [60].

## 4. Conclusions and Outlook

Luminescent MOFs materials offer a promising platform for light-emitting diodes, chemical sensing, bioimaging and anti-counterfeiting codes. In the past decades, much attention has been focused on design of linkers and encapsulation of guest molecules instead of lanthanide metal-based MOFs for environment consideration.

Encapsulation of organic dyes into MOFs is a feasible and ingenious approach to construct pc-WLEDs, which combines the benefits from dyes and MOF structures. The porosity and crystallinity of MOFs are helpful to suppressing ACQ of dye molecules and thus improving both fluorescent intensity and quantum yield. Meanwhile, the organic dyes enrich the luminescent behaviors without sacrificing the strength of MOFs. Although the warm white light can be generated by encapsulating fluorescent dyes in luminescent MOFs with high performance, the luminous efficiency is still low. In addition, organic dyes leakage, stability and unsuitable size hinder the extensive application of this method. According to the previous study, most reported dye@MOFs are synthesized based on currently available organic dyes or MOFs. From fundamental views, it is necessary to develop novel organic dyes and MOF structures considering factors such as topology, luminescence, charge transfer and stability. Moreover, in-depth research on mechanism behand should be devoted in order to provide instructions for material design and synthesis. For the purpose of industrialization and commercialization, the stability of phosphors, including photo-stability and thermal stability is of vital significance, while currently the reports on the stability of dye-encapsulated MOF phosphors are still rare. It is predictable that more effort will be devoted to investigate the stability of LMOF materials.

While the development of luminescent MOFs for WLEDs is still in infancy, it is evident that the future of WLEDs based on MOF is bright, not only because MOF structure provides various luminescence, but also because the low energy input can reduce the carbon footprint. There is still a long way ahead in achieving commercially available MOF-based WLEDs. In the coming decades, chemists and material scientists will work more closely to develop novel stable and high efficiency single phase white light emitting phosphors for WLED fabrication.

## Figures and Tables

**Table 1:** Structural information about organic ligands of MOFs.

MOF	Organic Ligand	Ref.
ZJU-28	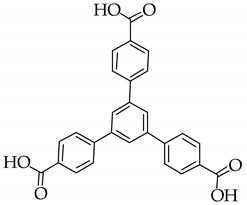	[34]
Bio-MOF-1	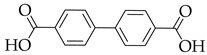	[35]
UiO-66	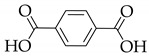	[36]
ZIF-8	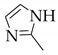	[36,37,38]
NKU-114	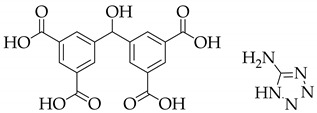	[40]
LIFM-WZ-6	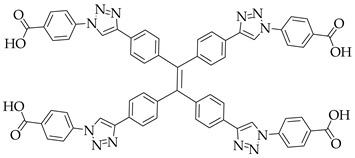	[42]
EuBPT TbBPT	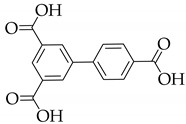	[45]
[Zn_4_OL_2_·*x*DMF]_n_	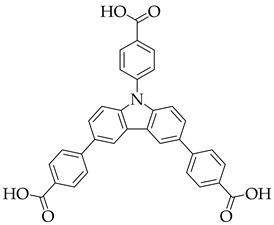	[50]
HSB-W1	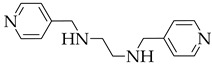	[51]
[Cd_7_(SO_4_)_6_(tppe)_2_] (2DMF·2H_2_O) [Zn_2_(npd)_2_(tppe)](2DMF·3H_2_O)	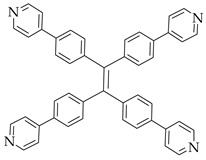	[54]
PCN-128W	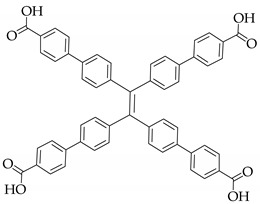	[55]
PCN-921	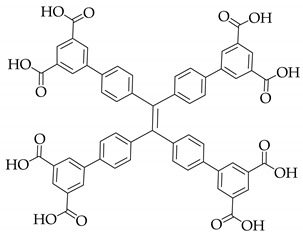	[56]
HNU-49	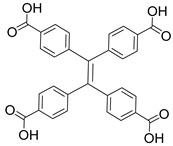	[57]

**Table 2:** Key parameters for white LEDs with dye-encapsulated MOFs as phosphors.

Dye-Encapsulated MOF Materials	CIE (*x*,*y*)	CCT(K)	CRI	QY (%)	Ref.
ZJU-28⊃Cou-6/R6G/R101	(0.34, 0.32)	4446	88	82.9	[34]
R6G@ZIF-8	-	-	--	63.1	[36]
ZIF-8⊃pm546/pm605/SRh101	(0.465, 0.413)	2642	85	-	[37]
NKU-114@DSM/AF/9-AA	(0.3402, 0.3365)	5148	85.41	-	[40]
CD-MOF⊃7-HCm@FL@RhB	(0.35, 0.32)	-	-	-	[41]
ZJU-28⊃DSM/ AF	(0.34, 0.32)	5327	91	17.4	[49]
[Zn4OL2·xDMF]n⊃DCM/C6	(0.32, 0.31)	6186	91	39.4	[50]
HSB-W1⊃DCM/C6a/CBS-127	(0.31, 0.32)	6638	90	26	[51]
DSM@PCN-128W	(0.34, 0.33)	5525	79.1	21.2	[55]

## Data Availability

Not applicable.
